# Contraceptive sterilisation: private practice, tubal ligation and vasectomy in twentieth-century Australia

**DOI:** 10.1017/mdh.2025.10015

**Published:** 2025-07

**Authors:** Tiarne Barratt-Young, Alison Bashford

**Affiliations:** University of New South Wales, Sydney, New South Wales, Australia

**Keywords:** Voluntary sterilization, Contraception, Family planning, Tubal ligation, Vasectomy

## Abstract

Surgical sterilisation practices significantly increased in contraceptive capacity as the twentieth century unfolded. Despite this prolific uptake, sterilisation is markedly absent from histories of birth control and family planning and instead has remained addressed within histories of eugenics and coercion. The purpose of this article is twofold: firstly, to demonstrate a voluntary, contraceptive history of sterilisation that is distinct from, though connected to, involuntary and eugenic sterilisation; and secondly, to explain the integral role that individual doctors and their private practice played in the rise of contraceptive sterilisation in twentieth-century Australia. Through a combination of archival material and oral history interviews with twentieth-century practitioners of tubal ligation and vasectomy, this article reframes the history of surgical sterilisation, situating it firmly within the history of birth control.

By the 1980s, sterilisation practices – tubal ligation and vasectomy – had become the most common method of birth control for married women in Australia, accounting for over 38 per cent of contraceptive use in this group, rivalled only by the oral contraceptive pill which accounted for 24 per cent of married women’s contraceptive use in 1986.^1^ A decade later, survey results included both married and unmarried women, which saw 22 per cent of women relying on sterilisation and 26 per cent of women relying on oral contraceptive pills as their primary method of birth control. In the three decades since the 1990s, there has been a downward trend in the use of tubal ligation and oral contraceptives, but an upward trend in vasectomy, long-acting reversible contraceptives (LARCs) such as intrauterine (contraceptive) devices (IUDs), implants, and injections, and male condoms.^2^ In regional areas of Australia, in particular, the uptake of LARCs has been prolific. They are quicker and easier to administer than sterilisation, and require less frequent medical intervention than short-term contraceptives.^3^ In spite of these significant changes, sterilisation remains a key contraceptive choice, representing the only long-term option for men, and the only non-hormonal, long-term option for women (excluding copper IUDs). Yet sterilisation is markedly absent from histories of birth control and family planning and has instead remained situated within histories of eugenics and coercive sterilisation.^4^

The problematic history of involuntary sterilisation is both undeniable and ongoing. Throughout the twentieth century, in both Australia and around the world, millions of sterilisation procedures occurred without full and complete consent.^5^ There was a vast spectrum of circumstances in which they were performed, from paternalistic doctors who believed they were acting in their patients’ best interests, to legalised, punitive sterilisation.^6^ In Australia, women and girls with disabilities have been the most frequent victims of involuntary sterilisation. This ongoing socio-legal issue has received increasing attention and has provoked calls to outlaw the practice since the 1990s.^7^ Around the world, men and women became embroiled in this history against their will, and this phenomenon has – quite rightly – been the subject of extensive historical analysis.^8^

Yet historians’ attentiveness to the sinister history of involuntary sterilisation in its many forms has obscured the parallel history of voluntary, consensual, contraceptive sterilisation, a phenomenon minimised within histories of birth control.^9^ Over the course of the twentieth century, both male and female sterilisation procedures were socially, culturally, medically, and legally transformed. What had been infrequent, inaccessible, and covert operations in the 1920s and 1930s, by the 1970s and 1980s had become widespread, popular, and readily accessible. This was made possible through a combination of changing social and religious attitudes around sex, gender, sexuality, and parenthood, advances in surgical technology, and an ever-increasing public demand for safe, reliable, long-term contraception in the contexts of the second wave feminist movement, universal Australian public healthcare, and mounting concerns around the long-term safety of oral contraceptives.

It is notoriously difficult to separate voluntary and involuntary sterilisation. The legal debates, frequently overlapping actors, and often-blurred lines around consent only accentuate the interconnected nature of this history.^10^ There is, nonetheless, considerable value in considering these histories separately – even if fully extricating them is not possible. Historian of medicine Ian Dowbiggin has argued that ‘the history of the sterilization movement is the untold story of the twentieth-century birth control movement, more important than the history of the pill and rivalling the significance of the history of abortion’.^11^ This article follows through on this claim, redressing an oversight within the history of birth control, and recovering the history of voluntary surgical sterilisation as a contraceptive choice.

This article analyses the rise in popularity and accessibility of sterilisation in the Australian context, paying particular attention to the role that individual doctors and private practice played in the proliferation of both tubal ligation and vasectomy as methods of voluntary birth control. Doctors were by no means the only actors in this story. Indeed, the patient perspective of men and women, usually middle class, usually white, who had the means to access non-essential private healthcare, is just as important, though beyond the scope of this article.^12^ Yet the surgical nature of sterilisation meant that practitioners played a unique role in regard to accessibility, their medical training inadvertently rendering them the clinical gatekeepers of permanent contraception.

This history has remained obscure and difficult to access precisely because of the closed world of private practice: methodologically, private practice source material is far more difficult to access than institutional or state records. The oral histories undertaken here are the sole record for significant mid- to late twentieth-century practitioners, while rare patient records provide insight into earlier twentieth-century practice. This article focuses on the experiences of six Australian doctors: Norman Haire (1892–1952), Victor Hugo Wallace (1893–1977), Stefania Siedlecky (1921–2016), Bruce Errey (b.1931), Barbara Simcock (b.1935), and Ian Stewart (b.1943), whose careers between them spanned the twentieth century. Two of these case studies are based on archival material, and four are based on oral history interviews and supplemented with their personal archival material.^13^

Of these six doctors, Haire, Wallace, and Siedlecky undertook their medical training in the early twentieth century. A graduate of the University of Sydney in 1915, Dr Norman Haire was a pioneering sexologist and prominent member of the Eugenics Education Society.^14^ In 1919, he departed Australia, continuing his practice in London’s Harley Street, before returning in 1940. His background in surgical gynaecology and his interest in the ‘rejuvenating’ properties of vasectomy meant that he was uniquely situated to perform both male and female sterilisation procedures, if he so chose.^15^ While Haire supported and performed hereditarily motivated sterilisations in both London and Sydney, his public commentary does not indicate that he was an early advocate of contraceptive sterilisation, rather the contrary.^16^ This is surprising, given his well-publicised sexual liberalism. Even so, this commentary provides rare insight into the regulatory role of individual practitioners in the interwar years.

Graduating shortly after Haire, Dr Victor Hugo Wallace was a radical general practitioner (GP) and a prominent member of the Eugenics Society of Victoria. He entered private practice in 1928, where he provided patients with birth control and contraceptive advice and began performing clandestine vasectomies in 1934. Around the time Wallace launched his private vasectomy practice, Dr Stefania Siedlecky commenced her medical training. From a low socio-economic background, Siedlecky was awarded a bursary to study medicine at the University of Sydney. From the 1940s to the 1980s, Siedlecky fought for women’s increased access to all forms of reproductive healthcare and discreetly performed contraceptive tubal ligations, both in private practice and in public hospitals throughout her career. These doctors approached sterilisation through a set of informal and internally determined rules that were then passed on to students, residents, and registrars, thereby shaping the next generation of practitioners.

Errey, Simcock, and Stewart were all educated in the mid-twentieth century, and although they inherited these older ideas, the changing social context in which they operated allowed them to pursue careers in contraceptive sterilisation more openly. Dr Bruce Errey was one of Australia’s most prolific vasectomists. Vasectomy was his passion as well as his livelihood, and he proudly ‘boasts’ that a total of 30,040 vasectomies were performed between 1970 and 2007.^17^ In 1970, vasectomy presented a unique opportunity for general practitioners, and Errey’s private clinic added an element of surgical prestige and an impact to his career that would have otherwise eluded him as a general practitioner. Errey’s contemporary, Dr Barbara Simcock, was equally pioneering, if less enthusiastic, establishing the first public outpatient vasectomy clinic in Australia in 1972 in conjunction with Family Planning New South Wales (NSW).^18^ Graduating in the late 1960s, Dr Ian Stewart pursued a career in obstetrics and gynaecology, specialising in laparoscopic surgery, including tubal ligation. In the early 1970s he was offered an obstetric position in Wagga Wagga in rural NSW, where he spent the remainder of his career providing surgical contraception in a town where he reported that tubal ligation has remained controversial well into the twenty-first century, morally opposed by the region’s strong Catholic community, though this is not representative of contemporary Australian attitudes more generally.

This article begins with a brief technical explanation of tubal ligation and vasectomy to highlight the differential difficulties of these surgical procedures. It then moves on to the rise of tubal ligation, in which the careers of Haire, Siedlecky, and Stewart are analysed to detail the transformation of tubal ligation, focussing on developments in surgical technology, the legal history of sterilisation, gynaecological gatekeeping, the introduction of the pill, and the changing social context of the 1960s. Finally, Haire, Wallace, Errey, and Simcock’s careers are analysed to explore the rise of vasectomy services in Australia, shaped by changing attitudes around marriage and the family in the 1950s, and a surprising connection to the Family Planning Association in India. Steering away from the well-known, early twentieth-century public discussion of eugenic sterilisation, this article instead uncovers the overlapping world of private practice and contraceptive sterilisation, demonstrating the interconnected, yet distinct aspects of this history.

## Tubal ligation and vasectomy procedures

Tubal ligation, known in lay terms as ‘tying the tubes’, is the surgical procedure most commonly employed to achieve female sterilisation and describes an operation that blocks a woman’s fallopian tubes, rendering her permanently sterile.^19^ Tubal ligation procedures are comprised of two main surgical components: methods of approaching the fallopian tubes and methods of occluding the fallopian tubes.^20^ Methods of approach fall into three categories: transvaginal, transcervical, and transabdominal.^21^ Transvaginal approaches were popular in the 1960s and 1970s due to their time efficiency, but are no longer recommended, while experimentation with transcervical techniques was only just beginning in the 1980s and was not incorporated into sterilisation practice until the twenty-first century. In contrast, abdominal methods of approach have been applied consistently throughout the twentieth century, and the technologies of laparotomy (open abdominal surgery), minilaparotomy (open surgery with a smaller incision), and laparoscopy (keyhole surgery) were the most frequently employed in practices of contraceptive sterilisation in twentieth-century Australia. Methods of occlusion also fall into three main categories: traditional surgical ligation, division, or excision, which involves tying, cutting, or removing a section of the tubes; mechanical devices such as clips and bands placed on the tubes and designed to block them; and non-surgical methods, the most successful of which has been electrocautery, and involves burning a section of the tubes in order to seal the passage.^22^

Vasectomy is the operation used to achieve male sterility, and in contrast to tubal ligation, it is a quick and straightforward outpatient surgical procedure. Vasectomy involves occluding the vas deferens (the tubes that carry sperm, commonly known as the vas or vasa) so that when a man ejaculates, it no longer contains any sperm, which prevents the possibility of conception occurring.^23^ Unlike the complex surgical nature of tubal ligation, vasectomy is a straightforward procedure – in the words of Australian vasectomy pioneer, Dr Barbara Simcock, ‘it’s not brain surgery!’^24^ The procedure consists of locating the vas deferens through a small scrotal incision under either local or general anaesthetic at the practitioner’s discretion, followed by occlusion of the vas via ligation, division, excision, or cauterisation. Although techniques of occlusion used in vasectomy are similar to those used in female sterilisation, the easily accessible location of the vas eliminates the need for complicated abdominal surgery, which has historically been a consistent technological barrier to achieving minimally invasive tubal ligation. Instead, access to the vas requires only a small scrotal opening, which is done via a double incision on either side of the scrotum, or a single incision in the middle of the scrotum, through which both vasa are reached. Throughout the twentieth century, the availability of vasectomy was often contingent on geographical location, the availability of resources, and the skill, knowledge, and training of the practitioner.

The developing surgical techniques and technology of tubal ligation and vasectomy are foundational to this history. From a medical perspective, as sterilisation procedures became increasingly time-efficient, cost-effective, and minimally invasive, doctors became more willing to perform elective surgery in contraceptive circumstances, which in turn increased the availability of surgical contraception. Tubal ligation became increasingly viable as the physical demands on the patient decreased with less invasive technology. In the case of vasectomy, the procedure was much simpler and could be performed by general practitioners rather than specialist surgeons, but attitudes around family, sex, and masculinity first needed to loosen before doctors felt confident in providing this service at all, or openly.^25^ As social attitudes toward contraception became more liberal, the legal ambiguity that had long been associated with sterilisation began to resolve, and when combined with new technology, the ‘sexual revolution’ of the 1960s, and the introduction of universal public healthcare under the Whitlam Government in the 1970s, the social barriers to contraceptive sterilisation gradually dissipated.^26^

## The rise of contraceptive tubal ligation in Australia

In the first half of the twentieth century, Australian women’s awareness of tubal ligation in a contraceptive capacity was growing, but neither surgical technology nor societal attitudes were receptive to it as an easily accessible option for long-term contraception.^27^ At this point, tubal ligation was only possible via the open abdominal surgery known as laparotomy and according to Norman Haire, it was ‘a major operation, necessitating two weeks in bed’, meaning that doctors were reluctant to operate unless it was medically necessary.^28^ In extreme circumstances, where a woman’s life would be endangered by further pregnancy, doctors would perform ‘therapeutic’ tubal ligations, but the gruelling nature of the surgery and the controversial status of contraception more generally meant that it was not recommended in non-therapeutic circumstances.^29^ In this context, there was extensive gatekeeping by the medical profession, fuelled by the belief that contraceptive tubal ligation – or indeed, contraception in general – was morally inappropriate, sinful and/or unlawful, regardless of the patient’s willingness, desire, or consent to undergo the physically demanding surgery. Legally, sterilisation was governed by the principles of assault, under which a person cannot legally consent to bodily harm, meaning that many doctors did not believe that a patient could consent to sterilisation.^30^

The legal status of sterilisation preoccupied much historical public debate and accordingly has also dominated historiographical enquiry. In the 1920s and 1930s, sterilisation was considered a matter of eugenic concern, its legality a constant question. This emphasis lingered into the 1960s in regard to contraceptive sterilisation, despite the retreat of eugenics from public debate, because it was largely the same actors involved: practitioners already wary of sterilisation’s unlawfulness except in therapeutic circumstances. For example, in the 1930s, both the Racial Hygiene Association (RHA) and the Eugenics Society of Victoria (ESV) advocated for the legalisation of sterilisation in the case of people who were considered to have any kind of hereditary or mental defect. Members of these groups believed that people in these circumstances should be able to access legalised, *voluntary* sterilisation, if they so desired – the RHA and ESV did not lobby for compulsory sterilisation.^31^ Yet legal ambiguity shrouded any sterilisation practice: ‘owing to there being no ruling in the New South Wales Laws, whether it [sterilisation] is legal or illegal, the position is very unsatisfactory’.^32^ In their exasperation, Australia’s eugenic societies turned to the British Medical Association (BMA) for guidance.^33^ They were advised that sterilisation was unlawful except in therapeutic circumstances – akin to therapeutic abortion.^34^ Thus, Australian doctors were reportedly ‘very chary’ of performing the operation in the early to mid-twentieth century, and it was largely deemed too controversial and legally risky to perform a tubal ligation when it was not therapeutically necessary.^35^

Indeed, sterilisation on eugenic grounds – either in a voluntary or involuntary capacity – was never successfully legislated in Australia.^36^ In 1928, William Ernest Jones, Victorian ‘Inspector General of the Insane’, was appointed to undertake an enquiry into mental deficiency in Australia, in which sterilisation of the ‘unfit’ was discussed. Jones found that: ‘sterilization will never be resorted to … until the economic pressure, arising from the increasing burden of lunacy and mental deficiency, has become very much more acute than it is at the present time’.^37^ In the states of South Australia, Tasmania, Victoria, Western Australia, and New South Wales, there were attempts to introduce mental deficiency bills during this time, some of which included hereditarily motivated sterilisation clauses, but none were successful due to their high cost and controversial content.^38^ During the interwar years, there was neither sufficient demand nor funding to support a push towards legalising eugenic sterilisation in Australia, and by the mid-twentieth century, public interest had moved on to contraceptive matters, though these earlier eugenic legal debates continued to have an ongoing influence on the perceived unlawfulness of contraceptive sterilisation.^39^

Despite his self-professed ‘very liberal views on the subject of sterilisation’, in 1928 Norman Haire argued that ‘[n]o surgeon of repute would perform it, for instance, in healthy young men and women who might ask for it simply because they wanted to be free to indulge in sexual intercourse without fear of pregnancy resulting’.^40^ By 1945, this position had not changed, and he insisted that he would not perform sterilisation procedures on patients wishing to ‘avoid parenthood as a matter of personal convenience’ or in order to ‘avoid a little trouble’, an attitude that upheld contemporary gendered expectations of parenthood.^41^ If Australian patients were to approach Haire for contraceptive sterilisation in the 1940s, he would allegedly, according to his articles in *Woman* magazine, advise them to wait and consider their decision for six months. Then, upon their return he would explain that he did not personally believe in performing sterilisation operations in contraceptive circumstances and suggested that they find another surgeon willing to do it, or to use other methods of birth control that would not impact their ability to have children at a later stage, should they change their mind.^42^ This was an attitude that doctors continued to hold throughout the twentieth century and demonstrates how integral individual doctors’ opinions and decisions were to the accessibility of contraceptive sterilisation.

Within this regulation of tubal ligation, age was a key consideration because doctors, upholding contemporary gendered limitations, believed that young women were not truly capable of deciding if they wanted any, or additional, children while still in their twenties. For instance, in 1948, Haire received a request from a female patient of 24 years of age asking for a tubal ligation. This woman already had one child and had undergone six illegal abortions prior to her consultation with Haire, attempting suicide when she had been unable to secure a seventh abortion. Yet, due to her young age, Haire refused to perform a tubal ligation as he ‘thought sterilisation unwise, as she might change her mind later on and *want* a baby’. Upon receiving this news, the patient ‘inquired rudely what right I [Haire] had to set myself up as a judge of whether the operation should be performed or not’. To which Haire replied that he was not judging whether it should happen or not, simply that he did not want to be the one to perform it.^43^

Unfortunately for this woman, few surgeons would perform a tubal ligation under these circumstances, or indeed, under any circumstances in 1948. And Haire was indeed making a judgment about whether the operation should take place or not, authorised by his elite surgical education, his gender, and the social context in which he operated. Throughout the 1940s, Haire continued to receive enquiries from women curious about the possibility of tubal ligation and how they could access this elusive procedure. While Haire was more than happy to share the technical surgical details of sterilisation, he would not provide practical advice about how these women could actually access tubal ligation procedures.^44^ Tubal ligation instead remained a covert, inaccessible operation for this generation of women, despite the increasing demand.

Contraception was not included in medical education curricula until much later in the twentieth century, long after Haire had ceased practising. This meant that subsequent generations of doctors received knowledge via apprenticeship, inheriting not just the surgical techniques, but authoritarian attitudes as well. This is evident in Stefania Siedlecky’s career as a gynaecologist who commenced her first residency at (Catholic) St Vincent’s Hospital in Sydney in 1937. Siedlecky recalled asking for further information about contraception as a medical student, and was told by various professors: ‘I’m here to teach you how to deliver babies, not how to prevent them!’ or that, ‘flood, famine, and disease will see to it that the world is not overpopulated’, which combined, represented the extent of the instruction she received.^45^ Indeed, none of the doctors included in this case study received any kind of formal education about surgical or non-surgical contraception; instead, they had to use their own initiative and learn from sympathetic colleagues upon qualification.

Siedlecky learned how to perform tubal ligation operations as a junior doctor by assisting senior gynaecologists at the Rachel Forster Hospital for Women and Children in Sydney in the 1940s. The Rachel Forster Hospital was significant because it was a hospital for women, staffed by women, that prioritised women’s health.^46^ Never solely an obstetrics and gynaecological hospital, by the late 1930s and early 1940s, gynaecological specialists were employed, but not for the provision of contraception. This was banned at this particular hospital, prohibited by the conservative views of senior staff – this conservatism is an ongoing barrier to contraception in many Australian contexts.^47^ However, tubal ligations could be, and were, undertaken at the discretion of individual gynaecologists if they deemed it therapeutically necessary. If a woman had undergone two or more caesareans, for example, further pregnancy was considered dangerous to her health, and a third caesarean would often be accompanied by a tubal ligation.^48^ In this context, Siedlecky was taught that it was only appropriate to perform tubal ligation if a woman’s age, multiplied by the number of children she had, resulted in a number over ninety, ideally closer to one hundred.

Stefania Siedlecky: There wasn’t any age requirements, but we had a few sort of odd ideas. One of them was, if you were 30 and you’d had 3 kids, you could have a sterilisation. Then if you were 25, you’d have to have had 4 kids to add up to 90.

This was rule-of-thumb knowledge, ‘just one of those things’.^49^ Despite later criticism of such entrenched practice in her book *Populate and Perish*, Siedlecky maintained that throughout her career, she refused to sterilise childless women in their twenties as she believed that they would later come to regret the decision. Haire’s earlier ‘doctor knows best’ attitude extended into later generations, even amongst liberal female practitioners such as Siedlecky, also an early provider of abortion services.^50^

After completing her gynaecological training at the Rachel Forster Hospital, Siedlecky returned to her hometown, the Blue Mountains suburb of Blackheath, west of Sydney, then a rural area, making this practice all the more controversial. Here, she opened her private practice in 1949, in which she provided women’s healthcare, including contraceptives and tubal ligation, offering valuable access to these services for women who would have otherwise faced geographical barriers. Similarly to Haire, Siedlecky initially showed restraint in performing tubal ligations, describing a ‘terrible operation’ which was associated with significant pain and discomfort because it consisted of ‘this great big cut’ that ‘took a couple of weeks to heal’.^51^ The development of mini-laparotomy and later laparoscopic technology was, in her opinion, the most significant change to her methods – interestingly, exceeding in importance any change to the legal status of the procedure. This technique is what enabled female sterilisation to move from open abdominal surgery to minimally invasive keyhole surgery, which was ground-breaking in relation to the efficiency of tubal ligation procedures, both in terms of reduced operating time and reduced recovery time, ultimately influencing doctors’ decisions to more readily perform this surgery in a contraceptive capacity.^52^

In a synchronistic turn of events, these technological developments in gynaecological surgery coincided with both the introduction of the pill and the ‘sexual revolution’ of the 1960s, facilitating rapidly changing social mores which resulted in a transformation of both attitudes and practices of tubal ligation.^53^ For example, Siedlecky recalled a significant increase in the frequency of the requests she received from women seeking to undergo contraceptive tubal ligation in the 1960s. In her view, women who had previously felt too embarrassed to discuss contraception with their doctor in the 1950s were given confidence by the introduction of the pill and were now empowered to ask for tubal ligations. ‘No one ever talked about it [contraception] before the pill, it never appeared in the paper, it changed the whole world as far as contraception was concerned’.^54^ As sex and reproduction became increasingly culturally (and biologically) separated, women became empowered to expect access to reliable and effective birth control in the 1960s. Contraception came to be considered a basic right, rather than an elusive privilege.

Set against the backdrop of women’s liberation, rapid population growth, fears of global resource shortages, and diminished moral opposition to birth control, the medical community began to support greater access to contraceptive sterilisation. Tubal ligation began to move away from being a covert operation that was practised in relative secrecy, or at least privacy, behind closed clinic doors, to something that women actively and successfully pursued as their preferred method of contraception. The social status of contraceptive sterilisation was thus changing in the 1960s, and it was in this era that government subsidies emerged, the Australian women’s health movement working with state governments and Family Planning clinics to improve women’s access to reproductive healthcare.^55^ In short, tubal ligation had become a much less demanding surgical procedure for both the surgeon and the patient and contraception had become less censored and covert after the introduction of the pill and the rapidly changing social context of the 1960s, all of which facilitated a rise in patient requests for tubal ligation procedures in the second half of the twentieth century, with doctors now under increasing pressure to meet these demands.^56^

The centrality of legislation in histories of early twentieth-century eugenic sterilisation debates has fostered a somewhat misguided assumption that legislation was equally important in the history of voluntary contraceptive sterilisation.^57^ Instead, the lawfulness or unlawfulness of voluntary contraceptive sterilisation for couples wishing to permanently limit the size of their families has never been officially clarified in Australia. Indeed, a 1985 report found that ‘there are no specific legislative provisions regulating sterilisation in any State or Territory in Australia and there is a dearth of general case law on the subject’.^58^ Rather, sterilisation was regulated by understandings of assault and consent and was considered permissible if it was performed for a ‘generally approved social purpose’.^59^ For much of the twentieth century, it was left to doctors to determine if non-therapeutic sterilisation constituted an approved social purpose, which ‘rest[ed] not on any immutable principle but on changing values in the community’.^60^ Therefore, no legislative change was required, and the practice was instead governed by public opinion. In this sense, patients collectively challenged medical authority in their demand for surgical contraception. As birth control became more acceptable, doctors could then be confident that contraception would be considered an ‘approved social purpose’ should they ever encounter any legal redress. Moving with the times, in 1970, the *British Medical Journal* updated its advice and confirmed that sterilisation operations were now legal in all circumstances except when considered to be ‘plainly injurious to the public interest’, for example, when done to facilitate a lifestyle of sexual promiscuity.^61^ This was followed by a recommendation from the Australian Law Reform Commission that sterilisation be nationally legalised. However, this legislation never eventuated, ultimately deemed unnecessary in light of the widespread socio-political acceptance of sterilisation.^62^ Thus, while there is a significant legal history of sterilisation in Australia regarding the coerced sterilisation of women and girls with disabilities, most notably Marion’s Case in the early 1990s, with regard to voluntary contraceptive sterilisation it is the absence of statutory or case law, or even policy regulation, that has enabled the practice.^63^

In the late 1960s, the safety of the pill came into question and was the subject of considerable negative attention in relation to both blood clots and long-term viability.^64^ While this alone does not account for the significant increase in demand for contraceptive sterilisation in the 1970s, it did raise issues about the need for long-term, reliable contraception for women who had completed their families, but still had many fertile years ahead of them. Women who had been using the pill throughout the 1960s were now faced with the question of ongoing contraception.

An increasing number of married couples desire permanent protection against the possibility of pregnancy. Their life style is satisfactory to them and they do not want any more children. Furthermore, they wish to avoid the possible undesirable side effects of oral contraceptives. The constant remembering to take the pill may become irksome.^65^

For those couples who sought permanent contraception rather than pregnancy spacing, tubal ligation and vasectomy had become increasingly realistic and appealing options, and by the 1970s, there was an explosion in the popularity of contraceptive sterilisation. In the case of tubal ligation, it was a dual transformation of technology and social attitudes. Technology influenced doctors’ decisions to perform the surgery, while the broader socio-political context of the 1960s and 1970s influenced women’s decisions to request the surgery. Together, this dispelled any lingering legal ambiguity.

Undergoing medical training in the 1960s, the next generation of doctors, including Ian Stewart, were primed to meet this new public demand and deliver widespread surgical contraception in the 1970s. Similar to Siedlecky, Stewart recalled that the introduction of the laparoscope was one of the most significant events of his career, utterly transforming his practice of tubal ligation.^66^ Indeed, within the medical profession, the popularisation of tubal ligation in the 1970s was directly attributed to this development in technology.^67^ This is an intriguing aspect of the history of tubal ligation, because patients themselves were rarely, if ever, in the 1970s and 1980s, provided enough surgical information about the procedure to even be aware that they were undergoing laparoscopic surgery. Instead, they were told only the bare minimum (permanency and recovery time), and as a result, the patients interviewed for this research did not recall this technological development.^68^

For medical professionals in this era, spousal consent was sometimes considered to be more important than informed patient consent. This lingered into the 1980s, and all doctors interviewed for this research, and Wallace in archival materials, indicated that they required spousal consent to perform both tubal ligation and vasectomy. This arose from a fear that disgruntled spouses could take legal action against the operating doctor if the procedure occurred without their knowledge, a concern fuelled by British reports of spousal legal action in the 1960s.^69^ However, in a gendered double standard, it was often easier for men to obtain a vasectomy without their wife’s consent, than for women to do the same with tubal ligation. For example, Errey recalled a male patient who wished to be sterilised in secret due to his wife’s religious beliefs, a reasoning that Errey accepted.^70^ In contrast, a female patient interviewed for this research expressed outrage that she required her husband’s permission for a tubal ligation in 1983.^71^ Equally, practitioners went to great lengths to ensure that men had not been coerced by their wives to undergo vasectomy, whereas such caution did not extend to women undergoing tubal ligation at their husbands’ request.^72^ Spousal consent eventually fell to the wayside as individual practitioners began to view it as unnecessary or inappropriate, Simcock and Stewart respectively describing it as ‘ridiculous’ and ‘demeaning’.^73^

Throughout the twentieth century, the practice of surgical contraception provoked an ongoing struggle between doctors’ and patients’ authority when it came to access to sterilisation procedures, as each party claimed a unique knowledge, either of their personal reproductive needs and choices, or of the surgical procedures required to realise those choices. By the mid-1970s, in the context of the second wave of feminism, some women were vehemently disputing medical authority in reproductive health, explaining the outrage expressed at the requirement for a husband’s consent in the 1980s.^74^ By the early 1980s, Australian women were largely in a position to request tubal ligation regardless of their age, marital status, or parity, and eventually without the consent of their husbands, making permanent contraception widely available for the first time.^75^ This demonstrates the distinct and significant role of tubal ligation within the history of the twentieth-century birth control movement, and these case studies emphasise the unique role that individual doctors’ decision-making played in this availability.

## The rise of contraceptive vasectomy in Australia

Like tubal ligation, vasectomy was an elusive, yet sought-after surgical procedure in the early twentieth century. In this context, the prevalence and accessibility of contraceptives were steadily increasing, yet male surgical contraception was highly problematised except in certain circumstances. Norman Haire believed vasectomy to be an ‘easy and harmless method of rendering men infertile without diminishing their sexual desire and potency’; however, it appears that he would only perform the procedure if he believed that it would be of therapeutic, rejuvenating, or eugenic benefit.^76^ Thus, while he had been practising vasectomy in London in these circumstances since the 1920s, there is no evidence that he was an early practitioner of contraceptive vasectomy. Yet, since much of the history of contraceptive sterilisation took place in private practice and given the absence of Haire’s patient records, there is a distinct possibility that Haire maintained a public-facing anti-contraceptive stance on sterilisation, while performing these procedures in private. Though we will never know the details of Haire’s private practice, his public stance on contraceptive vasectomy in the first half of the twentieth century indicates, at the very least, that there was increasing public demand for this minimally accessible procedure.

We know much more about the private practice of one of Haire’s Melbourne-based contemporaries. Victor Wallace was a radical general practitioner and a pioneering birth control advocate who surreptitiously provided his patients with both sexual counselling and contraceptive vasectomy services from the 1930s to the 1970s. A quiet early advocate, Wallace began performing the procedure in his private rooms in Melbourne in 1934, yet he did not speak of this practice publicly for another four decades, when he finally published on the subject in 1973.^77^ Indeed, as late as 1967, Wallace felt unable publicly to declare his long-term affiliation and history with contraceptive vasectomy, demonstrated in his response to a colleague’s *Medical Journal of Australia* article, which he chose to respond to privately, rather than publish in the journal as was common practice.^78^ Had Haire lived into old age, it is curious to think what he may have revealed in the rapidly changing social context of the 1960s and 1970s, given the length of time that it took Wallace to feel comfortable publicly sharing the details of his contraceptive sterilisation practice, despite his prominent role in the Eugenics Society of Victoria and the establishment of the first birth control clinic in Victoria, the Women’s Welfare Clinic located in the Melbourne suburb of Fitzroy.

Wallace is best known for his work in the provision of contraceptives, the birth control survey he conducted in the 1940s, and, more recently, his provision of sexual counselling in the first half of the twentieth century.^79^ However, Wallace was also one of Melbourne’s first known vasectomy providers, an aspect of his colourful career which has received surprisingly little attention to date. Wallace performed approximately 350 vasectomies in his private clinic, of which just over 200 patient records have survived and are now held in the Wallace papers at the University of Melbourne Archive. This makes Wallace one of the earliest documented providers of contraceptive vasectomy in Australia and represents a significant contribution to the history of the twentieth-century birth control movement. These records, though minimal in their detail, show a clear demand for contraceptive vasectomy from men from all walks of life – from butchers to Royal Australian Air Force (R.A.A.F.) pilots, from married to unmarried men, from men in their twenties to men in their fifties – and establish an early twentieth century history of vasectomy in this capacity during an era which was previously thought to be characterised only by eugenic sterilisation. Though a prominent member of the Eugenic Society of Victoria, Wallace considered his private vasectomy practice to be separate from the Society’s eugenic goals: an interesting point to note when attempting to separate these histories.^80^

Wallace began his vasectomy practice slowly, only gradually gaining confidence, and from 1934 until 1949 he recorded only 38 vasectomy patients: of these men, one sought the rejuvenating properties of vasectomy, three listed eugenic and hereditary concerns as their motivation for sterilisation, and the remaining 34 men’s procedures were ‘purely contraceptive’.^81^ Though few, these surviving patient records show a clear preference for and occurrence of contraceptive vasectomy, rather than eugenic vasectomy, during this period. Although isolated examples of rejuvenation and heredity such as these did occur on occasion, overall, it was contraception that drove the demand for Wallace’s vasectomy services. From the surviving records, we are able to discern that patients travelled from all around Australia, one patient even travelling from New Zealand, to access the service that Wallace was providing in the first half of the twentieth century.^82^ This gives a sense of the limited number of known vasectomy providers during this era, as well as the difficulty patients had in gaining reliable information about exactly how they could access a vasectomy. If doctors such as Wallace had not chosen to go against social convention and meet this public demand, then this service would have remained impossible to access.

The peak of Wallace’s vasectomy career was the 1950s, which aligns with broader social attitudes of the period around sex and sexuality, in which it was primarily men who assumed contraceptive responsibility.^83^ During the Second World War, increased concern about venereal disease, extramarital affairs, and divorce, creating an environment where sex and morality became more openly discussed. Escalating divorce rates in the post-war years were the context in which companionate marriage came to be emphasised: a sexual relationship based on mutual satisfaction was perceived to be its essence, leading to family stability, and even social order.^84^ Expectations of masculinity underwent significant change.^85^

Anxiety over unwanted pregnancy was often a barrier to couples achieving an ideal mutually satisfying sexual relationship and ensuing and elusive familial stability, and this was one of the reasons that propelled men to seek Wallace’s vasectomy services in the 1950s. For example, one patient’s letter explained that ‘We find the constant precautions are proving too much for our nerves and we feel that we should now be free of worry and be able to pursue a happy sexual liaison’.^86^ In this instance, the possibility of pregnancy was preventing this couple from achieving a companionate marriage, and as is clear from Wallace’s surviving sexual counselling records, this was an ideal that people strove to achieve. There was also a rise in vasectomy requests from men wishing to prioritise their wives’ physical and mental health in line with understandings of masculinity during this period, in which men were aspirationally responsible for their wives’ mental and social wellbeing. For example, one patient wrote that ‘I am requesting a vasectomy operation for the reason that I feel that my present family of three boys is quite sufficient for my wife and I to cope with without impairing her health, and for the happiness of all concerned’.^87^ Wellbeing of the entire family was beginning to be connected to the provision of vasectomy, and Wallace was happy to comply, strongly believing that contraception was a positive benefit: he saw no reason for married couples not to use contraception as they saw fit.^88^

Somewhat controversially for the social context, the vast majority of Wallace’s patients simply did not want any more children. Yet more controversially, Wallace sterilised men on these grounds alone and performed vasectomies on single, childless, and young men. If they were prepared to sign a letter of consent acknowledging the permanent nature of the procedure, then he was prepared to operate.^89^ As a doctor with surgical skill and willingness to operate, Wallace’s vasectomy practice was entirely self-regulated, and he could accept or refuse patients at his discretion. The controversial nature of contraceptive sterilisation was bound up in the ambiguous legality, which was tied to social attitudes rather than explicit regulations and explains why doctors such as Wallace were prepared to operate in private, but not comment publicly until the 1970s when social attitudes had become more receptive.

As we have seen in regard to tubal ligation, prior to the 1960s and 1970s, contraceptive sterilisation was not considered socially acceptable and accordingly, many doctors feared litigation.^90^ However, as socio-medical attitudes became more accommodating and the public demand for surgical contraception increased, sterilisation quickly transitioned into social acceptability, and therefore an admissible medical practice.^91^ The gradual dispelling of legal uncertainty and increasing acceptance of contraceptive sterilisation within the medical community was articulated in the *Medical Journal of Australia* (MJA) throughout the 1960s and 1970s.^92^ Driven by a popular demand for knowledge, the editors of the MJA finally broke their silence on sterilisation when they published a feature article in 1963 showcasing a variety of contemporary views on the ethics and legality of these practices in a purely contraceptive capacity.^93^ The editors erred on the side of caution and advised that sterilisation should still be considered illegal. Yet they were met with mixed responses, some colleagues supporting a continued rejection of surgical contraception, others hoping ‘that the Australian Medical Association [would] come down from its ivory tower and reconsider its attitude to sterilization operations’.^94^ This flurry of activity was followed by silence until 1967, when Perth-based doctor W.S. Haynes contributed an article advocating the benefits of contraceptive vasectomy, followed shortly after by an overview of eugenic sterilisation in Australia in the *MJA.* ^95^

Penney Lewis has traced the legal development of sterilisation in twentieth-century Britain, outlining how contraceptive sterilisation became legal for consenting adults without any changes made to existing law. Instead, it was a matter determined by changing public opinion, alongside increased support of the medical profession.^96^ The BMA’s stance on the legality of sterilisation was re-evaluated in 1960 as a direct result of the ‘change in the wind of opinion over recent years’.^97^ Once considered criminal assault and thereby illegal, the Medical Defence Union altered its position, declaring that: ‘An operation for sterilization is not unlawful whether it is performed on therapeutic or eugenic grounds or for other reasons, provided there is full and valid consent to the operation by the patient concerned’.^98^ In 1970, the *British Medical Journal* confirmed that practitioners should now consider sterilisation procedures to be permissible.^99^

In this new market of socially acceptable contraceptive vasectomy, pioneering general practitioners such as Bruce Errey and Barbara Simcock were able to make their mark on contraceptive sterilisation in Australia by opening clinics, training new doctors, and raising the profile and accessibility of vasectomy. During the late 1960s and early 1970s, public demand overshadowed previous controversy, and more and more Australians sought vasectomy services, yet there was a lack of existing infrastructure, which made these services difficult to access.^100^ It was choices made by individual doctors, such as Errey and Simcock, that slowly created a culture where vasectomy was readily available in Australia and ultimately transformed the contraceptive landscape.

Errey began performing vasectomies in Brisbane in April 1970, and within six months, men from around the state had begun to seek out his services. Indeed, he estimated that in the early years of his practice, he performed around 85 per cent of vasectomy operations undertaken in Queensland.^101^ In 2014, Errey recounted that he learned how to perform vasectomies over the phone when a colleague in Sydney described the procedure to him.^102^ This demonstrates the simplicity of vasectomy vis-à-vis tubal ligation, but also the extent to which individual general practitioners regulated this procedure amongst themselves. As doctors began to meet the increasing public demand, rates of use began to rise in correlation with ease of access and positive media attention. The popularity of vasectomy continued to grow. In 1971, the editors of the *MJA* remarked that ‘Vasectomy as a means of sterilization is not new, but it has rather suddenly become very topical … [and] the question of voluntary sterilization on eugenic grounds has been overtaken by the rapid growth in popularity and acceptance of sterilization for purely contraceptive reasons’.^103^ Errey was an enthusiastic vasectomist who happily met this public demand, making a point of celebrating every thousandth vasectomy he performed with an office party, the patient the guest of honour.^104^

Simcock was another practitioner of this era working to meet the increased demand for vasectomy in the 1970s. She worked as a doctor at the Family Planning Association (FPA) of NSW, providing clients with all manner of contraceptives and referring patients for both male and female sterilisation procedures from the mid-1960s onwards. Towards the end of the 1960s, Simcock noticed that requests for vasectomy referrals had started to gradually increase: once a rare occurrence, vasectomy requests began to occur monthly, and then weekly, until in 1971 the President of the FPA in NSW felt that the public demand had become sufficient to warrant a specialised, in-house vasectomy clinic which Simcock would run once she had received adequate training. Like Errey, Simcock contacted their south-Sydney colleague who had a reputation for performing vasectomies, but who did not wish to be interviewed for this project. She watched him perform the procedure in his private rooms (unlike Errey, who felt that a phone call was sufficient training) as part of her initial induction. But Simcock wanted more insight into how a public FPA vasectomy clinic could operate, and this is where her training took a unique and fascinating turn.

In February 1972, Simcock travelled to India to learn from the doctors who had become known for performing vasectomy as an effective method of family planning. The following excerpt from a 2013 oral history interview with Simcock is included here in its entirety as it provides rare insight into practices of and attitudes to vasectomy at the time, both in India and Australia, inaccessible in any other format.

*Barbara Simcock*: I used to go to a hospital down there in Bombay, and it was a special hospital only for Family Planning, and it was called The Family Planning Hospital. People would go there to have their tubes done on a daily basis, all very poor people … As for the vasectomy, they said: ‘You want to learn vasectomy? Sure! You’ll go out with our team.’

And the team consisted of the doctor, the nurse, and two untrained – I called them hookers. Their function was to get out onto the crowded road and snatch out suitable people. So, they wouldn’t pick up an old man, nor a young boy, they’d pick-up middle-aged men or men that they thought were right, and bring them in for a short interview with the nurse man, and if he thought he was a suitable candidate, then they’d get the doctor. All in a bus, a lovely big bus.

And apparently five children was the limit – you could not have a vasectomy unless you’d had five children. Of course, most of them had ten, eleven, twelve – so, if they said they only had four children, they’d be sent on their way, like throwing a little fish back into the sea. And again, you had to ask how many sons they had. So, if they had for instance, four daughters and one son, that wasn’t good enough – throw them back out into the sea.

And we’d park outside railway stations, Gateway of India, down by the harbour, but the railway station and the main bazaar was popular, and that was where you’d find people. And we did roughly seven to eight men per day, and I was there for two weeks. So, I got to see a lot, do a lot, learn a lot – gain confidence.^105^

Simcock’s experience was unique in the history of contraceptive sterilisation in Australia; she knew of no other doctor who received their vasectomy training in this manner. India was the first country to incorporate sterilisation into an organised family planning program and played a crucial role in the dissemination of vasectomy throughout the world in the second half of the twentieth century.^106^ Indian family planning has been the subject of extensive historical analysis and just a few short years after Simcock travelled to Bombay to learn how to perform vasectomies, India entered the Emergency from 25 June 1975–21 March 1977, a period of internal disturbance which in popular memory and historical scholarship has become synonymous with the widespread coercive sterilisation of millions of people.^107^ Such programs were already well underway at the time of Simcock’s visit, and sterilisation had been financially incentivised since 1959; the Emergency years simply the pinnacle of many years of increasingly forceful state policy around family planning.^108^ Indeed, the language used by Simcock, such as ‘hookers’ or people ‘snatching’, minimises the perceived need for consent, providing clear insight into the often-blurred line between voluntary and involuntary that so often characterises sterilisation. Simcock did not appear to be concerned about the incentivised vasectomy program with which she was briefly involved, but she used very different language to discuss her provision of vasectomies in Australia.

Errey and Simcock both went on to have successful careers as vasectomists over the next three decades, training new generations of practitioners and working towards the robust provision of vasectomy that is now available in Australia. Throughout this time, Errey and Simcock continued to perform the occasional procedure that was motivated by eugenic or hereditary concerns at their discretion, and they continued to act as gatekeepers of vasectomy when they considered a patient unsuitable. Errey, in particular, maintained a curious fascination with homosexuality and masculinity and would not operate on any man whom he perceived to be in any way effeminate. His rationales were idiosyncratic and diminishing. For example, he believed a man to be emasculated if he had too detailed a knowledge of his children’s birthdays or wife’s medical history and felt that men in these circumstances were not suitable candidates for vasectomy as their masculinity was already too compromised.^109^ Simcock, too, had patients whom she refused to sterilise if she felt they were too mentally unstable to make such a permanent choice, but did not express the same level of passionate, eccentric commitment as Errey. This demonstrates that long after the widespread popularity of vasectomy was realised, individual doctors maintained a significant stronghold over access to the procedure and continued to determine who could undergo contraceptive sterilisation throughout the remainder of the twentieth century. This provides significant insight into the authoritarian views of the medical profession on matters of sex, gender, disability, sanity, and the ability to consent and the powers they wielded. Yet, due to the work of practitioners such as Wallace, Errey and Simcock, vasectomy became a widespread, readily available method of long-term contraception. Vasectomy increased in popularity and availability from the 1980s onwards and currently accounts for 14 per cent of reported contraceptive use in Australia: a significant shift in the contraceptive landscape.

## Conclusion

There is a considerable lack in historical understanding of voluntary, contraceptive sterilisation and the way in which it was transformed from a covert, elusive, controversial practice to a widespread method of long-term contraception in a matter of decades. This article has provided insight into this transformation and has established that voluntary contraceptive sterilisation has a robust history, distinct from, yet intertwined with, that of coercive sterilisation. This history is characterised by the significance of private practice, the methods and views of individual doctors, and their power to regulate these procedures. The Haire and Wallace papers provide insight into sterilisation practices in the early twentieth century, while oral history interviews with Siedlecky, Errey, Simcock, and Stewart reveal the truly private, obscured nature of this history, information only accessible via their personal histories. This oral history has provided more information about the way in which sterilisation practices were transformed, how this surgical knowledge was passed down from practitioner to practitioner, and the complex relationship between Indian family planning and the rise of vasectomy services in Australia.

These doctors represented some of the earliest and most influential providers of contraceptive sterilisation, and the normalisation of tubal ligation and vasectomy would not have been possible if they had not been willing to meet the ever-increasing public demand. From the 1940s to the 1980s, both tubal ligation and vasectomy were entirely transformed, facilitated by the rapid social change witnessed in the mid-twentieth century, and made possible by the choices and actions of individual medical practitioners who took it upon themselves to provide surgical contraception, distinct from their eugenic practice of sterilisation. Their work significantly impacted the contraceptive landscape in Australia and reflected changing attitudes towards sex, gender, sexuality and the family throughout this period and into the twenty-first century, ultimately creating a more accessible and inclusive environment for contraceptive options and choices.

## Footnotes

1 United Nations Department of Economic and Social Affairs, Population Division (2022). *World Contraceptive Use 2022.*

2
*Ibid.*

3 In theory, contraceptive access is equitable throughout Australia, given that LARCs, sterilisation, and some short-term contraceptives are available through government subsidised healthcare. However, access to healthcare varies significantly based on geographical region, due to limited availability of service providers in regional and remote areas, creating health disparities across the country, especially for Indigenous Australians. Family Planning NSW, *Contraception in Australia 2005-2018*, Ashfield, Sydney: FPNSW, 2020; Australian Institute of Health and Welfare, *Rural and Remote Health*, 7 July 2022, https://www.aihw.gov.au/reports/rural-remote-australians/rural-and-remote-health.

4 For example, Frank Bongiorno , The Sex Lives of Australians: A History (Melbourne: Black Inc., 2012); Lisa Featherstone , Let’s Talk About Sex: Histories of Sexuality in Australia from Federation to the Pill (Newcastle: Cambridge Scholars Publishing, 2011).

5 For example, see: Randall Hansen and Desmond King , Sterilized by the State: Eugenics, Race, and the Population Scare in Twentieth-Century North America (New York: Cambridge University Press, 2013) 3–4 ; Alexandra Minna Stern , ‘Eugenics, Sterilization, and Historical Memory in the United States’, História, Ciências, Saúde-Manguinhos 23, Suppl. 1 (2016): 195–212 10.1590/S0104-5970201600050001128198932; Gemma Scott , ‘My Wife Had to get Sterilised’: Exploring Women’s Experiences of Sterilisation under the Emergency in India, 1975–1977’, Contemporary South Asia, 25 (2017): 70–84 .

6

Amy Jean-Jacques and Sam Rowlands , ‘Legalised Non-Consensual Sterilisation - Eugenics Put into Practice Before 1945, and the Aftermath. Part 1: USA, Japan, Canada and Mexico’, European Journal of Contraception and Reproductive Health Care, 23 (2018): 121–929624082 10.1080/13625187.2018.1450973; Amy Jean-Jacques and Sam Rowlands , ‘Legalised Non-Consensual Sterilisation - Eugenics put into Practice Before 1945, and the Aftermath. Part 2: Europe’, European Journal of Contraception and Reproductive Health Care, 23 (2018): 194–200 29671357 10.1080/13625187.2018.1458227; Ronli Sifris , ‘The Involuntary Sterilisation of Marginalised Women: Power, Discrimination, and Intersectionality’, Griffith Law Review, 25 (2016): 45–70 .

7 Susan M Brady and Sonia Grover, *The Sterilisation of Girls and Young Women in Australia: A Report Commissioned by Federal Disability Discrimination Commissioner Elizabeth Hastings*, 1997; Women With Disabilities Australia, Submission No 49 to Senate Standing Committee on Community Affairs, The Involuntary or Coerced Sterilisation of People with Disabilities in Australia, March 2013.

8

Mark A. Largent , Breeding Contempt: The History of Coerced Sterilization in the United States (New Brunswick, N.J.: Rutgers University Press, 2008); John Macnicol , ‘The Voluntary Sterilization Campaign in Britain, 1918-39’, Journal of the History of Sexuality, 2 (1992): 422–3811645868 ; Mathew Thomson , The Problem of Mental Deficiency: Eugenics, Democracy, and Social Policy in Britain c. 1870-1959 (Oxford: Clarendon Press, 1998); Stephen Garton , ‘Eugenics in Australia and New Zealand: Laboratories of Racial Science’, in Alison Bashford and Philippa Levine (eds.), The Oxford Handbook of the History of Eugenics (Oxford: Oxford University Press, 2010); Ross L. Jones , ‘The Master Potter and the Rejected Pots: Eugenic Legislation in Victoria, 1918-1939’, Australian Historical Studies, 29 (1999): 319–4210.1080/1031461990859610519400018; Sam Rowlands , ‘Sterilization of Those with Intellectual Disability: Evolution from Non-Consensual Interventions to Strict Safeguards’, Journal of Intellectual Disabilities, 23 (2017): 233–4929228865 10.1177/1744629517747162; Stephen Garton ‘“Liberty of the Nation”: Eugenics in Australia and New Zealand and the Limits of Illiberalism’, in Paul D. Stenhouse and J. H. Spencer (eds.), Eugenics at the Edges of Empire (Cham: Palgrave Macmillan, 2018); Paul A. Lombardo , Three Generations, No Imbeciles: Eugenics, the Supreme Court and Buck v. Bell (Baltimore: The John Hopkins University Press, 2008); C. Roth , ‘The Degenerating Sex: Female Sterilisation, Medical Authority and Racial Purity in Catholic Brazil’, Medical History, 4 (2020): 173–194 10.1017/mdh.2020.2PMC712026832284633.

9 Contraceptive sterilisation here refers to procedures which occurred in fully voluntary circumstances at the genuine instigation or request of a patient who was in pursuit of permanent contraception; also interchangeable with voluntary sterilisation. Coerced sterilisation here refers to procedures which occurred in an involuntary capacity, representing a spectrum of experiences, from government incentivised procedures to legalised punitive sterilisation, and everything in between; also interchangeable with involuntary sterilisation. Eugenic sterilisation here refers to procedures which could occur under either voluntary or involuntary circumstances but were defined by their hereditary motivations, that is, the desire to limit a person’s ability to procreate and potentially pass on hereditary medical conditions, or conditions so perceived at the time. Within this, a patient could voluntarily request to be sterilised (positive eugenics), or a governing body could insist on an involuntary procedure (negative eugenics). Therapeutic sterilisation is primarily an early twentieth-century medical term which refers to procedures which could occur under either voluntary or involuntary circumstances but were defined by their health motivations or medical benefits, i.e. if further pregnancy represented a danger to a woman’s life, then medical professionals would perform a tubal ligation, usually in conjunction with a caesarean section, which could be done with or without a woman’s knowledge/consent depending on the context.

10 The continuum between choice and coercion is analysed carefully in Johanna Schoen , Choice and Coercion: Birth Control, Sterilization, and Abortion in Public Health Welfare (Chapel Hill and London: The University of North Carolina Press, 2005).

11

Ian Dowbiggin , The Sterilization Movement and Global Fertility in the Twentieth Century (Oxford: Oxford University Press, 2008), 3–4 .

12 All patients interviewed in the course of this research underwent sterilisation between 1960 and 1985 and indicated that their tubal ligation or vasectomy was a positive and significant experience in their life; one which, for many, was a lifeline that had finally freed them from the anxiety of unwanted pregnancy. For further information on the patient experience of sterilisation, see: Tiarne Barratt, ‘Contraceptive Sterilisation: A History of Tubal Ligation and Vasectomy in Twentieth Century Australia, 1926-86’ (unpublished MPhil thesis: University of Sydney, 2015). Although it is beyond the scope of this article, it is important to note here that Indigenous and migrant experiences of sterilisation were profoundly different from their white counterparts, and Indigenous Australian women in particular were subject to non-consensual tubal ligation, as well as other non-surgical interventions such as the forced removal of children.

13 Norman Haire Collection, University of Sydney Rare Books Collection (henceforth NHC); Victor Hugo Wallace Collection, University of Melbourne Archives (henceforth VHWC); ‘The Normalisation of Contraceptive Sterilisation in post-war Australia’, Project No. 2013/534, approved by The University of Sydney Human Research Ethics Committee (HREC), 4 July 2013. These six doctors have not been ‘selected’ for study. Rather, few others are historically known to have practised in this way.

14

Diana Wyndham , Norman Haire and the Study of Sex (Sydney: Sydney University Press, 2012); Ivan Crozier , ‘Becoming a Sexologist: Norman Haire, the 1929 London World League for Sexual Reform Congress, and Organizing Medical Knowledge about Sex in Interwar England’, History of Science, 39 (2001): 299–329 11712572 10.1177/007327530103900303. See also: Caroline Rusterholz , ‘Testing the Gräfenberg Ring in Interwar Britain: Norman Haire, Helena Wright, and the Debate over Statistical Evidence, Side Effects, and Intra-Uterine Contraception’, Journal of the History of Medicine and Allied Sciences, 72 (2017): 448–6728973592 10.1093/jhmas/jrx044; Alison Bashford and Carolyn Strange , ‘Public Pedagogy: Sex Education and Mass Communication in the Mid Twentieth Century’, Journal of the History of Sexuality, 13 (2004): 71–99 .

15 See: Norman Haire , Rejuvenation: The Work of Steinach, Voronoff, and Others (London: George Allen and Unwin, 1924); Chandak Sengoopta , ‘Rejuvenation and the Prolongation of Life: Science or Quackery’, Perspectives in Biology and Medicine, 37 (1993): 55–66 .

16

Norman Haire , ‘Sterilization of Mental Defectives’, British Medical Journal, 2 (1922): 110 ; Norman Haire , ‘Birth Control’, in Norman Haire (ed.), Some More Medical Views on Birth Control (London: Cecil Palmer, 1928), 48 ; Wykeham Terriss, ‘A Doctor Looks at Life – Don’t Decide Lightly’, *Woman*, 30 April 1945, Box 2.25 (2), NHC; Norman Haire, ‘Sex Education – Sex and the Individual’, lecture no. 9, 13 November 1945, Adyar Hall, Box 2, no. 2.1 ‘Lectures’, NHC.

17 Interview with Dr Bruce Errey, 15 January 2014.

18 Interview with Dr Barbara Simcock, 24 July 2013.

19

Family Planning New South Wales (FPNSW), Contraception, Healthy Choices: A Contraceptive Clinic in a Book (Sydney: University of New South Wales Press, 2009), 121–9.

20

World Health Organization (WHO), Female Sterilisation: A Guide to Provision of Services (Geneva: World Health Organization, 1992), 16 .

21

Mark I. Evans et al., ‘Transabdominal Versus Transcervical and Transvaginal Multifetal Pregnancy Reduction: International Collaborative Experience of More Than One Thousand Cases’, American Journal of Obstetrics and Gynecology, 170 (1994): 902–88141224 10.1016/s0002-9378(94)70306-x; Michelle Whittum et al., ‘The History of Female Surgical Sterilization’, Journal of Gynecologic Surgery, 37 (2021): 6 .

22 WHO, *Female Sterilisation*, 16.

23 FPNSW, *Contraception, Healthy Choices*, 136–7.

24 Interview with Dr Barbara Simcock, 24 July 2013.

25

Gilbert Kasirsky , Vasectomy, Manhood, and Sex (New York: Springer, 1972), 40–2; Tony O. Pomales , ‘Men’s Narratives of Vasectomy: Rearticulating Masculinity and Contraceptive Responsibility in San Jose, Costa Rica’, Medical Anthropology Quarterly, 27 (2013): 23–42 23674321 10.1111/maq.12014.

26 Australian healthcare systems changed in the 1970s towards the provision of universal public healthcare via the introduction of Medibank under the Labor Whitlam government. Prior to this, the provision of healthcare in Australia was predominantly private, though supported by public subsidies with means-tested access to hospital and pharmaceutical care. An Australian Government not-for-profit insurer (Medibank) was introduced in the 1970s, under which all Australians could access free healthcare. The Liberal Fraser government abolished the scheme in 1981, only for it to reappear as Medicare in 1983. Since the 1990s, healthcare in Australia has continued to vacillate between public and private, with each change in government and is currently serviced by a combination of the two, albeit with increasing incentivisation for the uptake of private health insurance. Richard Scotton and Christine Macdonald, *The Making of Medibank* (University of New South Wales: School of Health Services Management, 1993); Stephen Duckett and Kristina Nemet , The History and Purposes of Private Health Insurance (Grattan Institute, 2019): 1–36 .

27 Wykeham Terriss, ‘A Doctor Looks at Life – Surgical Sterilisation’, *Woman*, 9 October 1950, Box no. 2.25 (1), NHC.

28 Wykeham Terriss, ‘A Doctor Looks at Life – Sterilisation Queries’, *Woman*, 23 April 1945, Box 2.25 (2), NHC.

29 Haire, ‘Sex Education’, Box 2, no. 2.1, NHC; Norman Haire, ‘The Prevention of Conception’, in Norman Haire et al. (eds.), *Encyclopaedia of Sexual Knowledge*, 2nd edn [1952] (London: Encyclopaedic Press, 1965), 289.

30 ‘Sterilization’, *The Medical Journal of Australia*, 2,7 (1963): 283.

31 ‘Special Meeting - Sterilization/Segregation’, 18 June 1935, in Racial Hygiene Association 1935 Annual Report, Mitchell Library Collection (henceforth MLC); W.E. Agar , ‘Eugenics and the Future of the Australian Population’, The Eugenics Society of Victoria (Melbourne: Brown, Prior, Anderson, 1939), 7 ; Angela Booth , ‘Voluntary Sterilization for Human Betterment: A Lecture delivered before the Society’, Eugenics Society of Victoria (Melbourne: Brown, Prior, Anderson, 1938), 5 .

32 ‘Special Meeting’, MLC, 3.

33 There is a close connection between British and Australian law, both governed by the tradition of common law, with a shared history and an ongoing trend of Australia turning towards Britain to ascertain relevant legal precedent. Therefore, in this circumstance, it was entirely understandable that a NSW body sought legal advice from its more established and better-resourced British counterparts.

34 Eugenics Society of Victoria, 1939 Annual Meeting Notes, 35, Box 3, VHWC.

35 Racial Hygiene Association, 1937 Annual Report, 4, MLC; Mark Finnane , ‘From Dangerous Lunatic to Human Rights?: The Law and Mental Illness in Australian History’, in Catherine Coleborne and Dolly MacKinnon (eds.), Madness in Australia: Histories, Heritage and the Asylum (Brisbane: University of Queensland Press, 2003), 23 .

36 Garton, ‘Eugenics in Australia and New Zealand’, 243–44.

37

W. Ernest Jones , Report on Mental Deficiency in the Commonwealth of Australia, Department of Health (Canberra: H.J. Green, Government Printer, 1929), 3, 17 .

38

Dave Earl , ‘A Group of Parents Came Together’: Parent Advocacy Groups for Children with Intellectual Disabilities in Post-World War II Australia’, Health and History, 13 (2011): 88 10.5401/healthhist.13.2.008422329261.

39 Jones, ‘The Master Potter and the Rejected Pots’, 339.

40 Haire, ‘Birth Control’, 48.

41 Terriss, ‘Don’t Decide Lightly’, Box 2.25 (2), NHC; Haire, ‘Sex Education’, Box 2.1, NHC.

42
*Ibid.*

43 Wykeham Terriss, ‘A Doctor Looks at Life – Sterilisation as a Last Resort’, *Woman*, 30 August 1948, Box 2.25 (1), NHC.

44 Terriss, ‘A Doctor Looks at Life’, 23 April 1945, Box 2.25 (2), NHC.

45 Interview with Dr Stefania Siedlecky, 2 September 2013.

46

Alison Bashford , ‘Separatist Health: Changing Meanings of Women’s Hospitals in Australia and England, c. 1870-1920’, in Lilian R. Furst (eds.), Women Healers and Physicians: Climbing a Long Hill (Lexington: University of Kentucky Press, 1997), 214–16.

47 Interview with Dr Stefania Siedlecky, 2 September 2013.

48
*Ibid.*

49
*Ibid.*

50 Siedlecky and Wyndham, *Populate and Perish*, 47.

51 Interview with Dr Stefania Siedlecky, 2 September 2013.

52

Jesse Olszynko-Gryn , ‘Laparoscopy as a Technology of Population Control: A Use-centered History of Surgical Sterilization’, in Heinrich Hartmann and Corinna R. Unger (eds.), A World of Populations: The Production, Transfer, and Application of Demographic Knowledge in the Twentieth Century in Transnational Perspective (New York: Berghahn Books, 2014), 148–50.

53 For the sexual revolution, including its critique in different contexts, see: Dagmar Herzog , Sexuality in Europe: A Twentieth Century History (Cambridge: Cambridge University Press, 2011); Nelly Oudhoorn , The Male Pill: A Biography of a Technology in the Making (Durham: Duke University Press, 2003).

54 Interview with Dr Stefania Siedlecky, 2 September 2013.

55

Toni Schofield , ‘Health’, in Barbara Caine et al. (eds.), Australian Feminism: A Companion (Melbourne: Oxford University Press, 1998), 125–7; Reiger, *Our Bodies, Our Babies*, 187–9.

56 Interview with Dr Stefania Siedlecky, 2 September 2013; Thomas Robertson , The Malthusian Moment: Global Population Growth and the Birth of American Environmentalism (New Brunswick: Rutgers University Press, 2012), 157 .

57 Garton, ‘Eugenics in Australia and New Zealand’; Jones, ‘The Master Potter and the Rejected Pots’.

58 This report provides another example of the interwoven histories of voluntary and involuntary sterilisation, the report aimed at clarifying the situation around allegedly voluntary sterilisation of women with disabilities, its findings also applicable in a contraceptive capacity for women without a disability. Lesley Vick and Frank Webster , ‘A Report on Voluntary Sterilization with Special Reference to Minors and Women who are Intellectually Disabled’, Clinical Reproductive Fertility, 3 (1985): 99–106 11658716.

59 Finlay and Sihombing, *Family Planning and the Law*, 95–9.

60
*Ibid.*, 97.

61 ‘Legality of Sterilization’, *British Medical Journal*, 1 (1970): 704–5.

62

H. A. Finlay and Sandra GlasBeek , Family Planning and the Law in Australia: Part A, Primary Family Planning Legislation (Sydney: Family Planning Association of Australia, 1973), 28–44 ; Terry Buddin , ‘The Elusive Right to Voluntary Sterilisation: A Statutory Panacea’, Law and Social Welfare (Sydney: Australian Council of Social Service, 1976), 37 ; ‘Legislation on Abortion, Sterilisation and Contraception’, *Australian Law Reform Commission*, 83 (1977): 12.

63 Legal debate around the forced sterilisation of women and girls with disabilities is ongoing. See: Laura Elliot , ‘Victims of Violence: The Forced Sterilisation of Women and Girls with Disabilities in Australia, Laws, 6 (2017): 8 ; Ronli Sifris , ‘The Involuntary Sterilisation of Marginalised Women; Power, discrimination, and Intersectionality’, Griffith Law Review, 25 (2016): 45–70 .

64 ‘A Report from the Australian Drug Evaluation Committee: Oral Contraceptives and Thromboembolic Disease’, *The Medical Journal of Australia*, 1 (1970): 1267–9; ‘Growing Fears in U.S. Over the Pill’, *The Sydney Morning Herald*, 23 March 1969, 25; Barbara Seaman, *The Doctors Case Against the Pill – 25th Anniversary Edition* (New York: Hunter House, 1995), 9.

65 Victor Hugo Wallace, ‘The Development of Family Planning in Australia’, unpublished manuscript [1977], 80. Box 35, VHWC.

66 Interview with Dr Ian Stewart, 26 November 2013.

67

Warren R. Jones , ‘Female Sterilization’, The Medical Journal of Australia, 2 (1974): 209 4278790 10.5694/j.1326-5377.1974.tb70707.x.

68 This question of patient education raises important questions around informed consent, a legal concept that was taking hold in the United States in the 1970s but was only formalised in Australian law in the 1990s: *Rogers v. Whitaker* (1992) 67 A.L.J.R 47 (High Court of Australia). Prior to this, there had been an acknowledged conceptual conflict between the American, patient-oriented understanding of consent that was legally established in the 1970s and the British, doctor-oriented approach to consent that was legally established in the 1980s. In *Rogers v. Whitaker*, the high court ruled in favour of the American, patient-oriented approach and determined that it fell under the principles of negligence. However, what was left out of this ruling was the question of who decides how much and what information doctors must provide patients, or the parameters around patient education. Don Chalmers and Robert Schwartz , ‘Rogers v. Whitaker and Informed Consent in Australia: A Fair Dinkum Duty of Disclosure’, Medical Law Review, 1 (2) (1993): 139–5911660490 10.1093/medlaw/1.2.139.

69 ‘Legality of Sterilization’, *British Medical Journal*, 2,5211 (1960): 1510; ‘Legality of Sterilization’, *British Medical Journal*, 1,5698 (1970): 704–5.

70 Interview with Dr Bruce Errey, 15 January 2014.

71 [Redacted for anonymity.]

72
*Ibid.*

73 Interview with Dr Barbara Simcock, 24 July 2013; Interview with Dr Ian Stewart, 26 November 2013.

74

Gwen Gray , How Australia came to have a National Women’s Health Policy, International Journal of Health Services, 21 (1) (1998): 107–2510.2190/PCPL-8XA9-WKXU-D1A39493755.

75 Interview with Dr Ian Stewart, 26 November 2013.

76 Wyndham, *Norman Haire and the Study of Sex*, 104; Haire, *Rejuvenation*, 10; Haire, ‘Sex Education’, 14, NHC.

77

Victor H. Wallace , ‘Vasectomy’, Medical Journal of Australia, 1 (1973): 212 4694277 .

78 Letter from V.H. Wallace to W.S. Haynes, 26 May 1967, in response to ‘Vasectomy’, *Medical Journal of Australia*, 1967, 1, 1045. Box 5, ‘Medical Journals’, VHWC.

79 Lisa Featherstone, ‘Sexy Mamas?: Women, Sexuality and Reproduction in Australia in the 1940s’, *Australian Historical Studies*, 2005, 36, 238–42; Marilyn Lake, ‘Female Desires: The Meaning of World War II’, *Australian Historical Studies*, 1990, 24, 271, 284; Kerreen Reiger , The Disenchantment of the Home: Modernizing the Australian Family 1880-1940 (Melbourne: Oxford University Press, 1985), 118–20; Lisa Featherstone , ‘The Science of Pleasure: Medicine and Sex Therapy in Mid-twentieth-century Australia’, Social History of Medicine, 2018, 31, 445–61.

80 Wallace, ‘The Development of Family Planning in Australia’, 15–16, 35–7. Box 35, VHWC; Wallace, ‘The Eugenics Society of Victoria’, 216–17.

81 Vasectomy Patient Cards, Boxes 63 and 65, VHWC. However, no further details regarding the eugenic and hereditary specifications of these operations are available.

82 Wallace, ‘The Development of Family Planning in Australia’, 4–5, Box 35, VHWC; Patient History Cards, Box 63, VHWC; Patient cards and letters, Box 45, ‘Miscellaneous’, VHWC; Box 63, ‘Patient History Cards’ and Box 65.A ‘Patient History Cards Concerning Vasectomy (1940-60)’, VHWC.

83 Until the mid-twentieth century, contraception was considered a male responsibility, during which time women’s involvement was the exception. It was only later, after the introduction of the pill, that contraception was reframed as a ‘woman’s question’. Therefore, a more realistic understanding of historic birth control practices becomes increasingly likely when men are factored into this equation and contraception is viewed as part of a series of gendered interactions and decisions, rather than when discussion is restricted to women’s experiences. Kate Fisher , Birth Control, Sex, and Marriage in Britain 1918-1960 (Oxford: Oxford University Press, 2006), 191–3, 238–40; ‘Patient History Cards’, Box 63, VHWC.

84 Featherstone, ‘The Science of Pleasure’, 448–50; Featherstone, *Let’s Talk About Sex*, 229–34.

85

Johnny Bell , ‘Putting Dad in the Picture: Fatherhood in the Popular Women’s Magazines of 1950s Australia’, Women’s History Review, 22 (2013): 905–6, 917; Mark Peel , ‘A New Kind of Manhood: Remembering the 1950s’, Australian Historical Studies, 27 (1997): 147 .

86 Vasectomy patient letter of consent, 25 February 1953, Box 65.A, VHWC.

87 Vasectomy patient letter of consent, 29 August 1951, Box 65.A, VHWC.

88 Wallace, ‘The Development of Family Planning in Australia’, 1–2, Box 35, VHWC.

89 Vasectomy Patient Cards, Box 65.A, VHWC. Though, of course, there was still dispute at this time as to whether patients could legally consent to sterilisation, explaining why Wallace kept his vasectomy practice out of the public eye.

90 Technically, the risk of litigation was higher prior to the 1960s; however, no practitioner in Australia or Britain was ever charged with assaulting a patient as a result of performing a vasectomy or tubal ligation procedure. The greater risk came from disgruntled spouses who did not approve of their partners’ decision to undergo sterilisation; ‘Legality of Sterilization’, *British Medical Journal*, 2 (1960): 1510; ‘Legality of Sterilization’ (1970): 704–5.

91

Penney Lewis , ‘Legal Change on Contraceptive Sterilisation’, The Journal of Legal History, 32 (2011): 313–15.

92 Throughout the 1960s and ‘70s, *The Medical Journal of Australia* received much correspondence from doctors throughout the country related to contraceptive sterilisation, discussing everything from personal opinion to an exchange of techniques, and the differences between tubal ligation and vasectomy.

93 ‘Sterilization’, *The Medical Journal of Australia*, 2 (1963): 283–5.

94 ‘Sterilization – Correspondence’, *The Medical Journal of Australia*, 2 (1963): 429, 516, 558.

95 ‘The Case for Eugenic Sterilization’, *The Medical Journal of Australia*, 2 (1967): 219–20.

96 Lewis, ‘Legal Change on Contraceptive Sterilisation’, 295, 306–8.

97 Finlay and Sihombing, *Family Planning and the Law*, 97; For a detailed account of the legal history of contraceptive sterilisation in Britain, see: Lewis, ‘Legal Change on Contraceptive Sterilisation’.

98 ‘Legality of Sterilization’ (1960), 1516.

99 ‘Legality of Sterilization’ (1970), 704–5.

100 For example, in 1970, a couple travelled more than 2,000 kms from Rockhampton, Queensland to undergo vasectomy in Wallace’s private rooms, in Melbourne, Victoria, because their local GP did not know where else to refer them to. Vasectomy patient record and accompanying letter of consent, 18 March 1970, Box 65.B, VHWC.

101 Interview with Dr Bruce Errey, 15 January 2014; Patient records from Bruce Errey Private Collection, 1970-1972 (henceforth BEPC).

102
*Ibid.* For analysis of the telephone and medical communication, see: Jeremy A. Greene , The Doctor Who Wasn’t There: Technology, History, and the Limits of Telehealth (Chicago: University of Chicago Press, 2022).

103 Voluntary Male Sterilization’, *The Medical Journal of Australia*, 1 (1971): 455.

104 Interview with Dr Bruce Errey, 15 January 2014.

105 Interview with Dr Barbara Simcock, 24 July 2013.

106 See, Sanjam Ahluwalia , Reproductive Restraints: Birth Control in India, 1877-1947 (Urbana: University of Illinois Press, 2008); Sarah Hodges , Contraception, Colonialism and Commerce: Birth Control in South India, 1920-1940 (Aldershot: Ashgate, 2008); Sarah Hodges (ed.), Reproductive Health in India: History, Politics, and Controversies (Hyderabad: Orient Longman, 2006); Mohan Rao , From Population Control to Reproductive Health: Malthusian Arithmetic (New Delhi: Sage Publications, 2004); Emma Tarlo , Unsettling Memories: Narratives of the Emergency in Delhi (London: Hurst, 2003).

107

Rebecca Jane Williams , ‘Storming the Citadels of Poverty: Family Planning under the Emergency in India, 1975–1977’, Journal of Asian Studies, 72 (2014): 471–92. For similar exchanges over sometimes counterintuitive global north/south directions, see: Jesse Olszynko-Gryn , ‘Technologies of Contraception and Abortion’, in Nick Hopwood , Rebecca Flemming and Lauren Kassell (eds.), Reproduction: Antiquity to the Present Day (Cambridge: Cambridge University Press, 2018), 535–52.

108

Matthew Connelly , ‘Population Control in India: Prologue to the Emergency Period’, Population and Development Review, 32 (2006): 629–67; Kaval Gulhati , ‘Compulsory Sterilization: The Change In India’s Population Policy’, Science, 195 (1977): 1300–5841329 10.1126/science.841329.

109 Petah Digby, ‘A Vasectomy Clinic: Observational Study’, paper presented at the Australian Anthropological Society Annual Conference, Sydney University, August 1978, 3, BEPC; Interview with Dr Bruce Errey, 15 January 2014.

